# Dynamic behavior and control analysis in a new chaotic three-tier supply chain system with a sinusoidal modelling uncertainty for resilient manufacturing networks

**DOI:** 10.1038/s41598-025-29907-1

**Published:** 2025-12-30

**Authors:** Julita Nahar, Kankan Parmikanti, Monika Hidayanti, Muhamad Deni Johansyah, Sundarapandian Vaidyanathan, Rameshbabu Ramar, Aceng Sambas, Chittineni Aruna

**Affiliations:** 1 Department of Mathematics, Universitas Padjadjaran, Jatinangor, 45363 Sumedang, Indonesia; 2 Centre for Control Systems, Vel Tech University, Vel Nagar, Avadi, Chennai, 600062 Tamil Nadu India; 3 Centre of Excellence for Research, Value Innovation & Entrepreneurship (CERVIE), UCSI University, Cheras, Kuala Lumpur, 56000 Malaysia; 4 Department of Electronics and Communication Engineering, V.S.B. Engineering College, Karur, 639111 Tamil Nadu India; 5 Faculty of Informatics and Computing, Universiti Sultan Zainal Abidin, Campus Besut, Kuala, 22200 Terengganu Malaysia; 6 Department of Mechanical Engineering, Universitas Muhamadiyah Tasikmalaya, Tamansari Gobras, Tasikmalaya, 46196 Indonesia; 7 Artificial Intelligence Research Centre for Islam and Sustainability (AIRIS), Universiti Sultan Zainal Abidin, Gongbadak, 21300 Terengganu Malaysia; 8 Department of Computer Science and Engineering, KKR & KSR Institute of Technology and Sciences, Guntur, 522017 Andhra Pradesh India

**Keywords:** Chaotic supply chain system, Nonlinear dynamics, Lyapunov exponents, Bifurcation analysis, Amplitude control, Offset boosting, Network resilience, Engineering, Mathematics and computing, Physics

## Abstract

This paper introduces a novel chaotic three-tier supply chain system (CSCS) that integrates both absolute function and sinusoidal nonlinearities into the classical Hamidzadeh model to enhance its dynamic complexity. The key improvement in the proposed model is that it exhibits higher Lyapunov exponent values (*l*_*1*_ = 0.2121) compared to the existing models, conforming stringer chaotic dynamics. Further, amplitude and location of the chaotic signal can be controlled in the proposed model. The proposed model captures the interactions among manufacturers, distributors, and retailers while exhibiting rich chaotic behaviors characterized through Lyapunov exponents, Lyapunov dimensions, and bifurcation analysis. Numerical simulations reveal improved chaotic intensity compared to existing CSCS models, with clear transitions between fixed points, periodic orbits, and chaos under parameter variations. To improve practical applicability, two control strategies are implemented: amplitude control, enabling systematic scaling of state variables without altering the chaotic nature, and offset boosting control, which shifts attractors in phase space while preserving system dynamics. Comparative analysis demonstrates the superior dynamic range and flexibility of the proposed model, offering valuable insights for designing resilient and adaptive supply chain networks under uncertainty.

## Introduction

Modern global supply chains are highly interconnected networks that link manufacturers, distributors, and retailers through intricate flows of materials, information, and capital^1^-^2^. These multi-tier systems operate across geographic, economic, and political boundaries, enabling the production and delivery of goods at unprecedented scales and speeds. Manufacturers are responsible for producing and assembling products, often sourcing raw materials and components from multiple countries^3^. Distributors act as intermediaries, managing inventory, transportation, and warehousing to ensure timely delivery to retailers, who in turn provide goods to end consumers^4^. The performance of each tier is interdependent; disruptions in one stage—such as a factory shutdown, transportation delay, or sudden demand surge—can propagate rapidly through the entire network^5^. This high degree of interconnection, while enhancing efficiency and responsiveness, also increases vulnerability to instabilities and amplifies the effects of uncertainties in demand, supply, and market conditions^6^.

Demand variability, lead time uncertainty, and market fluctuations are among the most critical factors that can destabilize supply chain operations^7^. Fluctuating customer demand, driven by seasonal trends, shifting preferences, or unexpected events, can cause sudden surges or drops in orders that ripple through the network^8^. Lead time uncertainty, stemming from transportation delays, production bottlenecks, customs clearance issues, or supplier reliability, further complicates planning and coordination between tiers^9^. Market fluctuations, including changes in raw material prices, currency exchange rates, and competitive pressures, introduce additional volatility into decision-making processes^10^. When these factors interact within a tightly coupled, multi-tier system, small disturbances can be amplified over time, leading to oscillations in production schedules, inventory levels, and delivery performance—a phenomenon often described as the bullwhip effect^11–13^.

Complex behavior in supply chains arises from the nonlinear interactions between their various components, feedback loops, and decision-making processes^14^. Even in well-planned systems, delays in information sharing, ordering policies based on forecasts, and batch processing of orders can introduce oscillatory patterns in production and inventory levels^15^. This often manifests as the “bullwhip effect,” where small fluctuations in end-customer demand are magnified as they move upstream through the supply chain, resulting in excessive inventory swings, underutilized capacity, and inefficiencies^16^-^17^. Nonlinear relationships—such as demand depending on price in a non-proportional manner or production capacity being constrained by variable factors—further increase the system’s unpredictability^18^. Under certain parameter conditions, these feedback-driven oscillations can transition into chaotic dynamics, where long-term behavior becomes highly sensitive to initial conditions, making accurate forecasting and control extremely challenging^19^-^20^.

The introduction of chaos theory into supply chain research has provided a powerful lens for understanding the unpredictable yet deterministic behavior that can emerge in these systems^21^. Chaos theory, originally developed in the context of nonlinear dynamical systems, demonstrates that even simple deterministic models can generate highly complex and irregular patterns when certain conditions are met^22^. In supply chains, feedback loops, time delays, and nonlinear decision rules can interact in ways that produce sensitive dependence on initial conditions—a hallmark of chaos^23^. This means that small changes in demand forecasts, production schedules, or transportation times can lead to disproportionately large and unexpected outcomes over time. Researchers have developed mathematical models that reveal how oscillatory behaviors in inventory levels and order rates can transition into chaotic regimes, making them inherently difficult to predict and control^24^. In recent years, many chaotic and hyperchaotic systems with unique dynamical features have been introduced, including discrete memristor hyperchaotic system^25^, 2D hyperchaotic map^26^, hidden multiscroll chaotic system^27^, and memristor system with coexisting attractors^28^.

Several studies have explored the application of nonlinear dynamics and chaos theory to supply chain systems, revealing the potential for complex and unpredictable behaviors in multi-tier networks. Early works by Forrester and Sterman demonstrated how feedback delays and ordering policies can generate oscillations and instabilities in inventory systems, laying the groundwork for later chaos-based analyses^29^-^30^. Hamidzadeh et al.^31^ introduced a three-tier CSCS modeled as a jerk system, capturing interactions between manufacturers, distributors, and retailers. Building on this, Johansyah et al.^32^ incorporated an absolute function nonlinearity, while Johansyah et al.^33^ introduced a sinusoidal nonlinearity to account for periodic market fluctuations, both enhancing the richness of the system’s dynamics. Beyond supply chains, chaos control and amplitude regulation techniques have been applied in various nonlinear systems, including financial models^34^ and mechanical oscillators^35^, offering valuable strategies for managing instability without destroying chaotic characteristics. Additionally, studies in nonlinear network systems^36^ have highlighted the benefits of combining chaos modeling with intelligent control, paving the way for adaptive responses to parameter changes.

The main contribution and novelty of this work as follows:

- We proposed a three-tier chaotic supply chain system combining absolute and sinusoidal nonlinearities for richer dynamics.
- We conducted comprehensive dynamic and comparative analysis showing improved complexity and controllability over existing models.
- We applied amplitude control and offset boosting to manage chaos without altering system nature.

This paper is organized as follows: Sect. 2 presents the mathematical formulation of the proposed three-tier chaotic supply chain system, incorporating both absolute function and sinusoidal nonlinearities. Section 3 provides a comprehensive dynamic analysis of the system, including Lyapunov exponents, Lyapunov dimensions, and bifurcation diagrams with respect to key parameters. Section 4 introduces the Amplitude Control mechanism, demonstrating how it regulates the magnitude of chaotic signals without altering the system’s inherent dynamics. Section 5 discusses the Offset Boosting technique, showing how it can shift attractors in phase space while preserving chaotic behavior. In Sect. 6, discusses the main results obtained in the manuscript. Finally, Sect. 7 concludes the paper by summarizing the main contributions, and suggesting directions for future research.

## A new chaotic supply chain model

In 2023, Hamidzadeh et al.^31^ described a Chaotic Supply Chain System (CSCS), which is modelled with the following jerk dynamics:

1 $$\begin{gathered} {{\dot {p}}_1}={p_2} \hfill \\ {{\dot {p}}_2}={p_3} \hfill \\ {{\dot {p}}_3}=a{p_1} - b{p_2} - {p_3} - p_{1}^{2} \hfill \\ \end{gathered}$$

Hamidzadeh CSCS model (1) represents a 3-tier supply chain network comprising (i) manufacturers ($\:{p}_{1}$), (ii) distributors ($\:{p}_{2}$), and (iii) retailers ($\:{p}_{3}$) with the route of transfer of the supply chain model from left to right. In the Hamidzadeh CSCS model (1), the financial constants $\:a$ and $\:b\:$designate the retailer satisfaction and distributor satisfaction of the manufacturer’s products (commodities) respectively.

In 2024, Johansyah et al.^32^ proposed a new Chaotic Supply Chain System (CSCS) by modifying the dynamics of the Hamidzadeh CSCS model (1). Explicitly, Johansyah et al. (2) added absolute function nonlinearity in the Hamidzadeh CSCS model (1) and stated their dynamics as given below:

2 $$\begin{gathered} {{\dot {p}}_1}={p_2} \hfill \\ {{\dot {p}}_2}={p_3} \hfill \\ {{\dot {p}}_3}=a{p_1} - b{p_2} - {p_3} - |{p_1}| - p_{1}^{2} \hfill \\ \end{gathered}$$

The 3-tier supply chain network given in the Johansyah CSCS model (2) has the same economic interpretation as the Hamidzadeh CSCS model (1).

In 2024, Johansyah et al.^33^ proposed another new Chaotic Supply Chain System (CSCS) by introducing a sinusoidal nonlinearity in the dynamics of the Hamidzadeh CSCS model (1). The sinusoidal nonlinearity in the Johansyah CSCS model represents the modelling uncertainty in the supply chain model. Johansyah CSCS model^33^ is stated by the following jerk dynamics:

3 $$\begin{gathered} {{\dot {p}}_1}={p_2} \hfill \\ {{\dot {p}}_2}={p_3} \hfill \\ {{\dot {p}}_3}=a{p_1} - b{p_2} - {p_3} - p_{1}^{2} - c\sin ({p_1}) \hfill \\ \end{gathered}$$

The 3-tier supply chain network given in the Johansyah CSCS model (3) has the same economic interpretation as the Hamidzadeh CSCS model (1).

In this research paper, we introduce a new 3-tier chaotic supply chain network by merging the dynamics given in the Johansyah CSCS model (2) and the Johansyah CSCS model (3). In other words, we add both an absolute function nonlinearity and a sinusoidal nonlinearity to the jerk dynamics given in the Hamidzadeh CSCS model (1). Thus, we state the new jerk dynamics for the 3-tier supply chain network as follows:

4 $$\begin{gathered} {{\dot {p}}_1}={p_2} \hfill \\ {{\dot {p}}_2}={p_3} \hfill \\ {{\dot {p}}_3}=a{p_1} - b{p_2} - {p_3} - p_{1}^{2} - c\sin ({p_1}) - q|{p_1}| \hfill \\ \end{gathered}$$

The absolute nonlinear term introduces piecewise, and non-smooth behavior, meanwhile sinusoidal nonlinearity produces periodic and smooth behavior, which helps to create chaos oscillations. The combination of two nonlinear effects enhances the complexity of dynamic behavior in the new model (4). The proposed integration of absolute and sinusoidal nonlinearities is motivated by the need to capture structured, periodic uncertainties inherent in real-world supply chains, such as seasonal demand fluctuations, production cycles, and scheduled maintenance.

The proposed model (4) represents a 3-tier supply chain network comprising (i) manufacturers ($\:{p}_{1}$), (ii) distributors ($\:{p}_{2}$), and (iii) retailers ($\:{p}_{3}$), the financial constants $\:a$, $\:b\:$and *c* designate the production adjustment rate, demand sensitivity, and retailer satisfaction of the manufacturer’s products (commodities) respectively.

The 3-tier supply chain network given in the new CSCS model (4) has the same economic interpretation as the Hamidzadeh CSCS model (1). Also, we let $\:P=({p}_{1},{p}_{2},{p}_{3})$ to denote the 3-D state of the new CSCS model (4). For $\:P\left(0\right)=\left(\text{0.5,0.5,0.5}\right)$ and $\:\left(a,b,c,q\right)=\left(\text{9,4.2,0.6,0.5}\right),$ the Lyapunov characteristic exponents (LCE) of the new CSCS model (4) can be calculated using Wolf algorithm with step size 0.1 and simulation time $\:T=1\times\:{10}^{4}$ seconds as follows:

5 $$l\_\left\{ 1 \right\}~={\text{ }}0.2121,l\_\left\{ 2 \right\}~={\text{ }}0,l\_\left\{ 3 \right\}~=~ - {\text{ }}1.2121$$

From the values of the LCE in (5), we deduce that the proposed 3-tier supply chain network model (4) is chaotic and dissipative with the maximal Lyapunov characteristic exponent (MLCE) given by $\:{l}_{\text{m}\text{a}\text{x}}=0.2121.\:$Moreover, Lyapunov dimension of the new CSCS model (4) is evaluated as follows:

6 $${d_L}=2+\frac{1}{{|{l_3}|}}({l_1}+{l_2})=2.1750$$

**Table 1:** Comparative analysis between the proposed model (4) and existing jerk models in literature.

CSCS Model	Lyapunov exponents	MLCE	Lyapunov dimension
Hamidzadeh model^31^	*l*_1_ = 0.1700, *l*_2_ = 0, *l*_3_ = -1.1700	*L*_max_ = 0.1700	2.1453
Johansyah et al.^32^	*l*_1_ = 0.1650, *l*_2_ = 0, *l*_3_ = -1.1650	*L*_max_ = 0.1650	2.1456
Johansyah et al.^33^	*l*_1_ = 0.1787, *l*_2_ = 0, *l*_3_ = -1.1787	*L*_max_ = 0.1787	2.1516
Proposed model	l_1_ = 0.2121, l_2_ = 0, l_3_ = -1.2121	L_max_ = 0.2121	2.1750

Table 1 presents the comparative analysis between the proposed model (4) and existing models in terms of their Lyapunov exponents and Lyapunov dimensions. The introduction of absolute function nonlinearity and a sinusoidal nonlinearity within Hamidzadeh model^1^ leads to notable improvement in the Lyapunov exponent values.

**Fig. 1:**
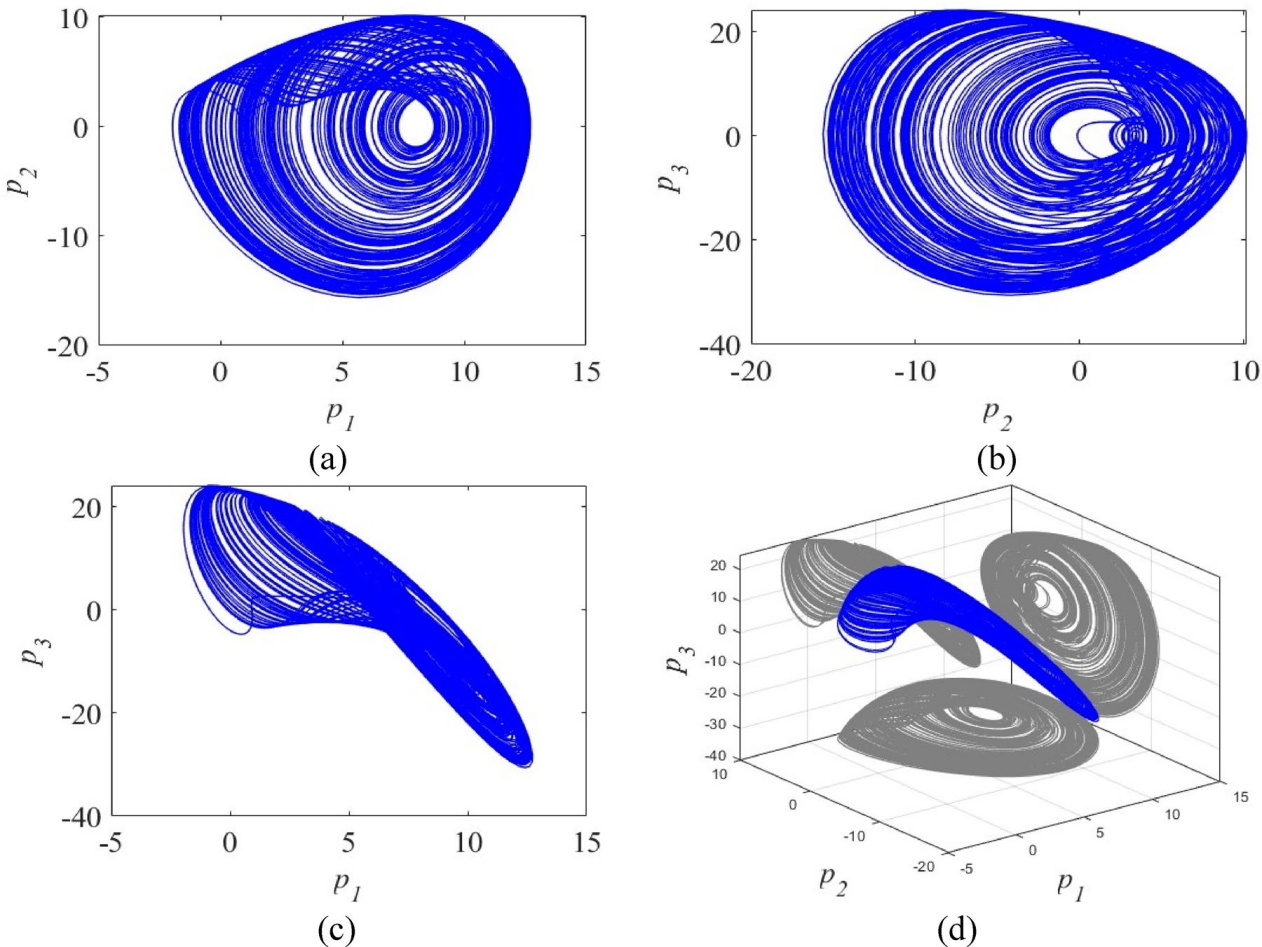
Phase portraits of the proposed system (4) in various 2D planes and 3D space.

The rest points of the new chaotic supply chain (CSC) model (4) are got by solving the following system of equations:

7a $${p_2}=0$$

7b $${p_3}=0$$

7c $$a{p_1} - b{p_2} - {p_3} - p_{1}^{2} - c\sin ({p_1}) - q|{p_1}|=0$$

We use the equations (7a) and (7b) to conclude that *p*_2_ = 0 and *p*_3_ = 0. Then Eq. (7c) simplifies to

8 $$a{p_1} - p_{1}^{2} - c\sin ({p_1}) - q|{p_1}|=0$$

For the chaotic case $\:\left(a,b,c,q\right)=\left(\text{9,4.2,0.6,0.5}\right),$ Eq. (8) has two roots

9 $${p_1}=0\,and\,{p_1}=8.8640$$

Therefore, there are two equilibrium points for the new CSC model (4) given by

10 $${P_0}=(0,0,0)\,and\,{P_1}=(8.8640,0,0).$$

**Fig. 2:**
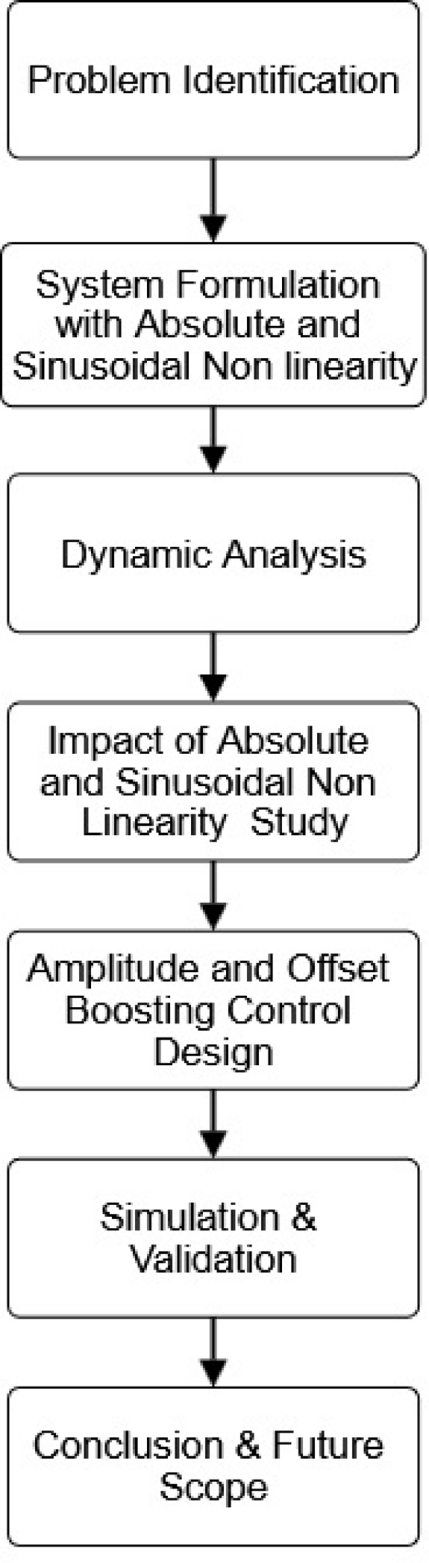
Research methodology flow chart.

The Jacobian matrix of the new CSC model (4) at ${P_0}$ has the eigenvalues $\:1.5148$ and $\:-1.2574\pm\:2.0563i.$ Hence, ${P_0}$ is an unstable saddle-focus equilibrium point for the system (4). Also, the Jacobian matrix of the new CSC model (4) at ${P_1}$ has the eigenvalues $\:-2.0315$ and $\:0.5157\pm\:1.9569i.$ Hence, ${P_1}$ is also an unstable saddle-focus equilibrium point for the system (4). The phase portraits of the proposed system (4) are given in Fig. 1. The research methodology flow chart is shown in Fig. 2.

## Dynamic analysis

The bifurcation diagram plays important role to understand the complex behavior of the nonlinear dynamical systems as the system parameters are changed. The bifurcation diagram reveals the system’s behaviors such as fixed point, periodic orbit and chaos with respect to the bifurcation parameters. The Lyapunov exponent (LE) spectrum is another important tool used to understand the sensitivity to initial conditions behavior which is the indication of chaos in the dynamical systems. In this section, we plotted the bifurcation diagrams and LE spectrum of the proposed system (4) with respect to all the system parameters using the initial conditions (0.5, 0.5, 0.5). The numerical simulations of the proposed chaotic supply chain system and bifurcation analysis were performed using the fourth-order Runge–Kutta (RK4) integration method with a fixed time step of 0.001 and a total simulation time of 300 units.

**Fig. 3:**
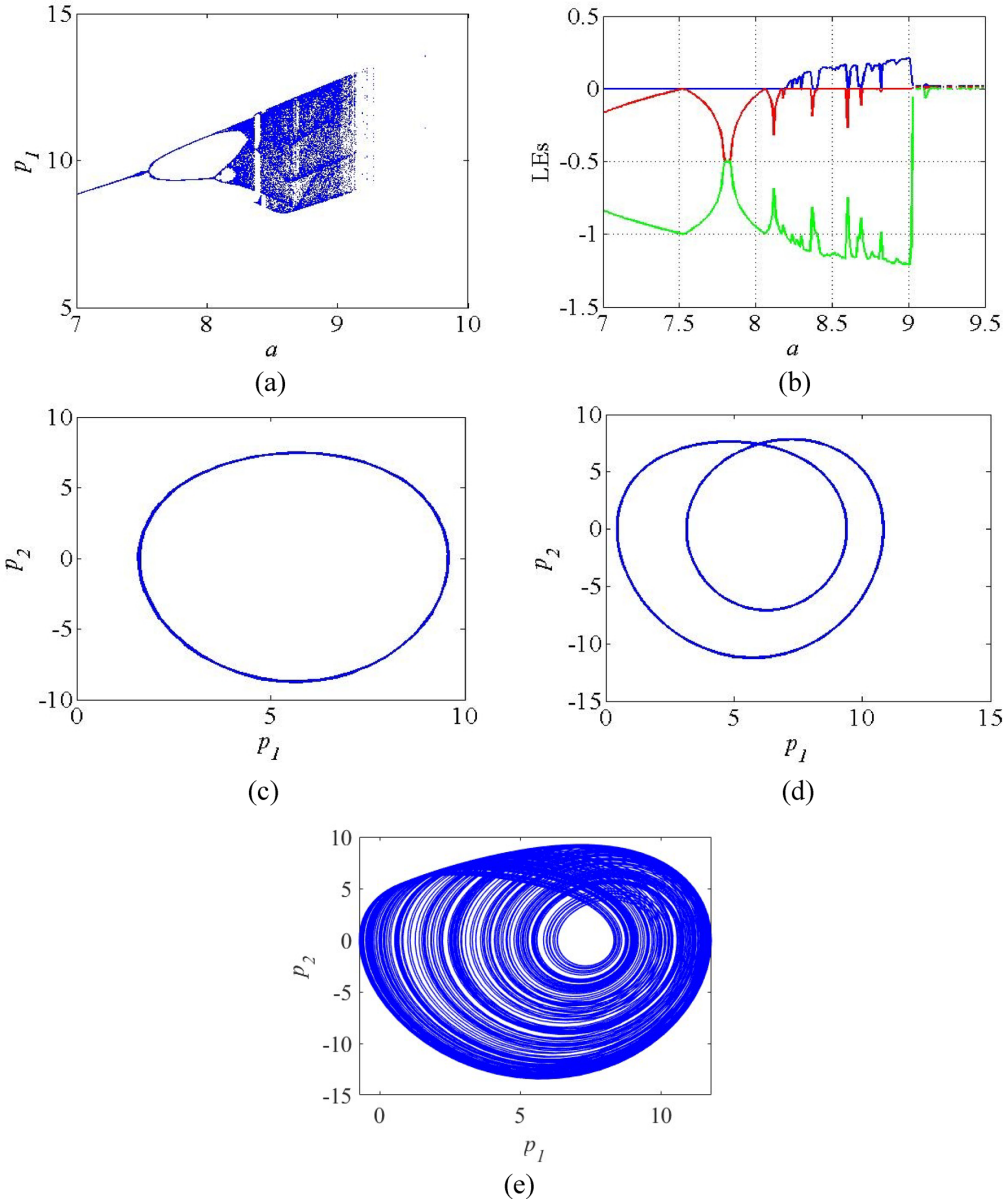
(**a**) Bifurcation diagram; (**b**) LE spectrum of the system (4) (**c**) Periodic orbit at *a* = 7.5; (**d**) Periodic attractor at *a* = 8; (**e**) Chaotic attractor at *a = 8.5*.

Figure 3 shows the bifurcation diagram and LE spectrum of the system (4) as the function the parameter *a*. It can be realized from Fig. 3a that the system’s dynamics change from fixed point to periodic orbits, and subsequently by chaotic oscillation as the parameter *a* varies with in the range *a* = 7 to *a* = 9.5. The system exhibits fixed points with in the region *a* = 7 to *a* = 7.5, as indicated by a single line, periodic orbits with in the region *a* = 7.6 to *a* = 8.2, characterized by two distinct lines. From *a* = 8.3 to *a* = 9, the system exhibits chaotic attractors, as evidenced by dense points in the bifurcation diagram. The corresponding LE spectrum for the parameter *a* are given in Fig. 3b in which the positive values of LE_1_ in the range *a* = 8.3 to *a* = 9 indicates the presence of chaos in the system. Beyond *a* = 9, all the LE values become zero. Figures 3c-e show the plots of periodic and chaotic attractors for various parameter region.

**Fig. 4:**
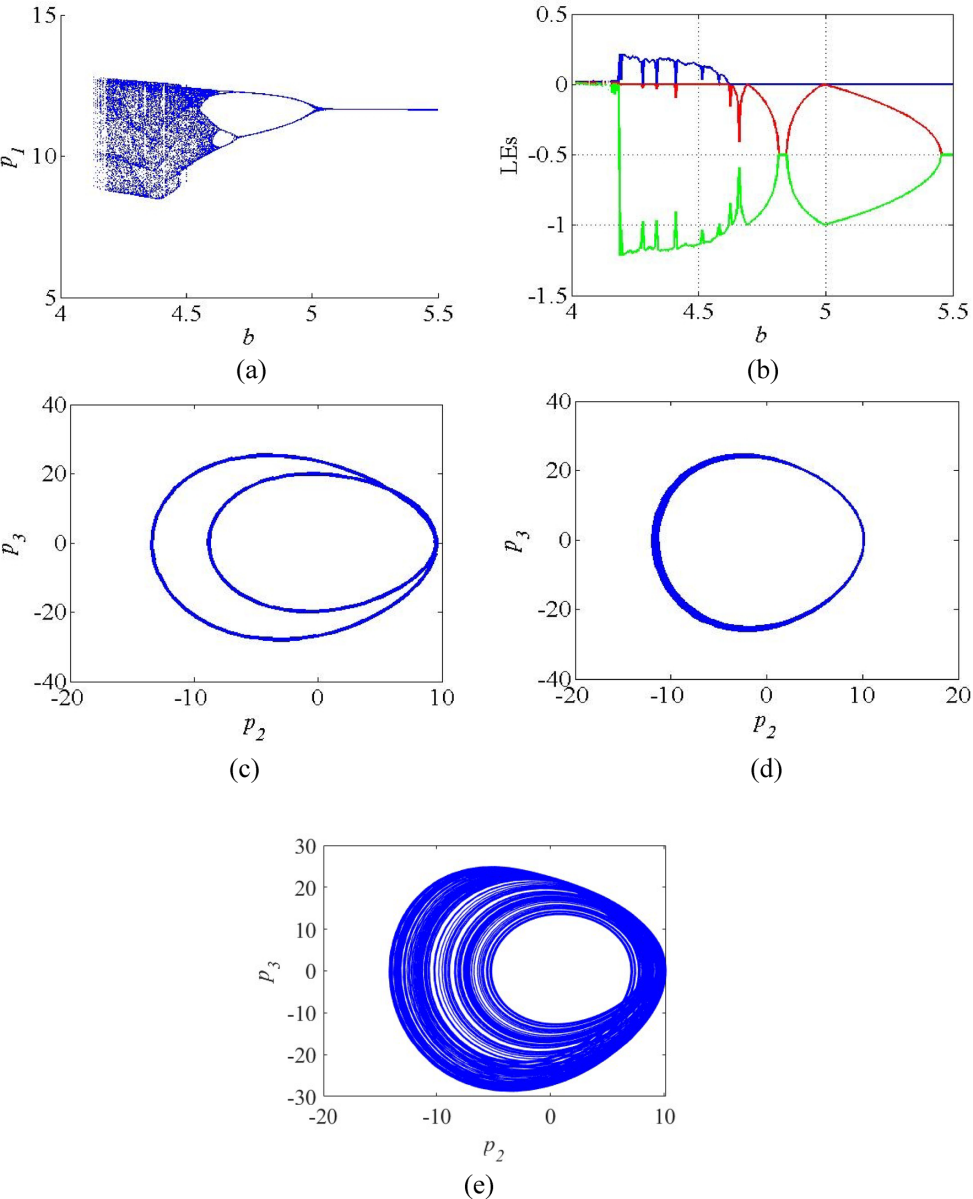
(**a**) Bifurcation diagram; (**b**) LE spectrum of the system (4); (**c**) Periodic attractors at *b* = 4.7; (**d**) Periodic orbit at *b* = 5; (**e**) Chaotic attractors at *b = 4.5*.

Figures 4shows the bifurcation diagram and LE spectrum of the system (4) as the function the parameter *b*. Under the parameter b, the system exhibits successive chaotic regions, periodic regions and subsequently stable points. Figure 4a, the bifurcation diagram indicates that the system has the following dynamics as the parameter varies in the range *b* = 4 to *b* = 5.5: chaotic dynamics in the region *b* = 4.2 to *b* = 4.55, characterized by dense regions, periodic dynamics in the region *b* = 4.6 to *b* = 5, and stable points in the region *b* = 5.1 to *b* = 5.5, shown in single line. The bifurcation results are validated by plotting the corresponding LE spectrum as given in Fig. 4b. For the regions 4.2 < *b* < 4.55, the spectrum exhibits LE_1_ > 0, LE_2_ = 0, LE_3_ < 0, which indicates the presence of chaotic attractors in the proposed system. Beyond *b* = 4.55, the system exhibits the periodic attractors and stable points, evidenced by LE_1_ = 0, LE_2_ < 0, LE_3_ < 0 in the LE spectrum. Figures 4c-e show the plots of period − 2 attractor, periodic orbit and chaotic attractors when *b* = 4.7, *b* = 5 and *b = 4.5* respectively.

**Fig. 5:**
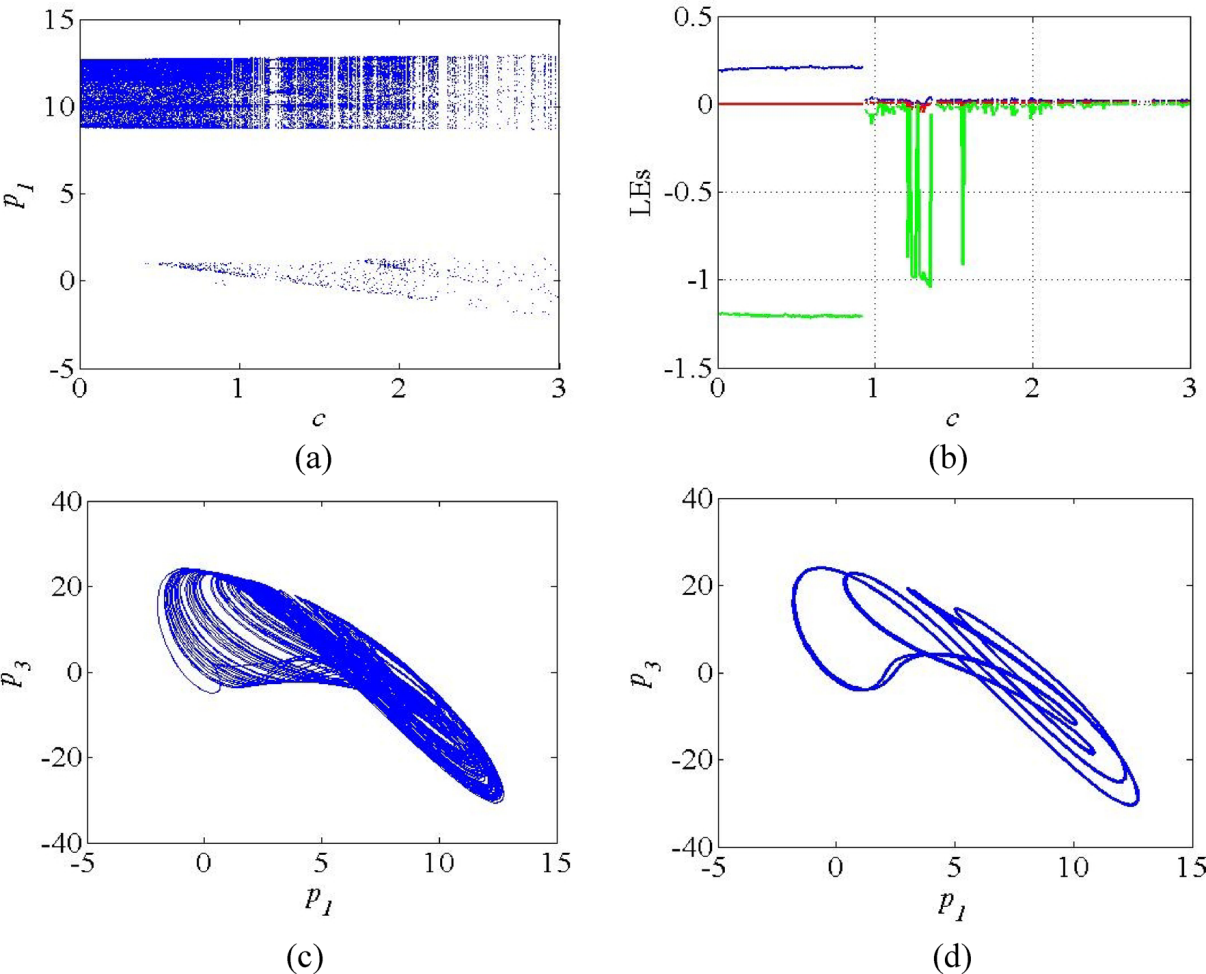
(**a**) Bifurcation diagram; (**b**) LE spectrum of the system (4); (**c**) Chaotic attractors at *c* = 0.8; (**d**) Periodic attractor at *c* = 1.3.

Figure 5 shows the bifurcation diagram and LE spectrum of the system (4) as the function the parameter *c*. Figure 5a reveals that the system exhibits chaotic regions for the lower values of *c* in the interval 0 < c < 0.95 and periodic regions in the interval 1 < c < 3. The corresponding LE spectrum for the parameter *c* is also given in Fig. 5b to validate the bifurcation result. The LE spectrum exhibits positive LE_1_ values in the interval 0 < c < 0.95, which indicates the sensitivity on initial conditions, the feature of chaos. Beyond *c* = 0.95, the system exhibits the periodic attractors evidenced by zero LE_1_ values in the LE spectrum. Figures 5(c-d) show the plots of period − 2 attractor and stable points when *b* = 4.7 and *b* = 5 respectively.

**Fig. 6:**
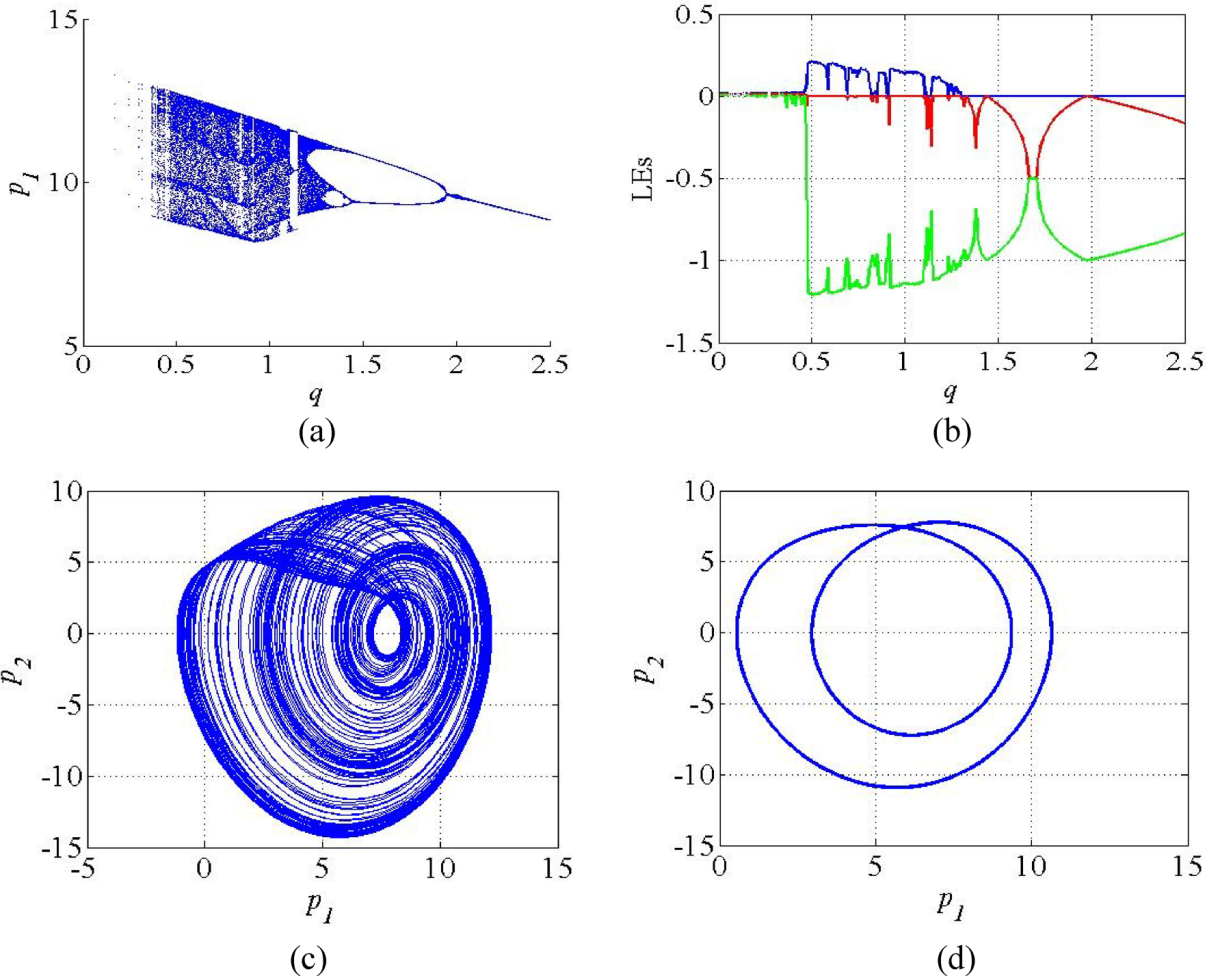
(**a**) Bifurcation diagram; (**b**) LE spectrum of the system (4); (**c**) Chaotic attractor at *q* = 0.8; (**d**) Periodic attractors at *q* = 1.6.

Figure 6 depicts the bifurcation diagram and LE spectrum of the system (4) for the system parameter *q* in the interval 0 < *q* < 2.5. In bifurcation diagram (Fig. 6a), reveals that the system exhibits successive chaotic, periodic and stable regions as the parameter *q* is changed in the interval 0 < *q* < 2.5. Initially, the system exhibits chaotic region in the interval 0.5 < *q* < 1.3, evidenced by dense region. After that the system exhibits periodic and stable regions in the interval 1.35 < *q* < 1.95 and 2 < *q* < 2.5 respectively. The corresponding LE spectrum plotted as given in Fig. 6b, where the positive LEs in the region 0.5 < *q* < 1.3 conforms the presence of chaos and sensitivity on initial conditions feature. For *q* > 1.3, LE_1_ drops to zero, indicating the periodic behavior in the system. The attractors in the chaotic and periodic regions of the system (4) are given in Fig. 6(c-d).

**Fig. 7:**
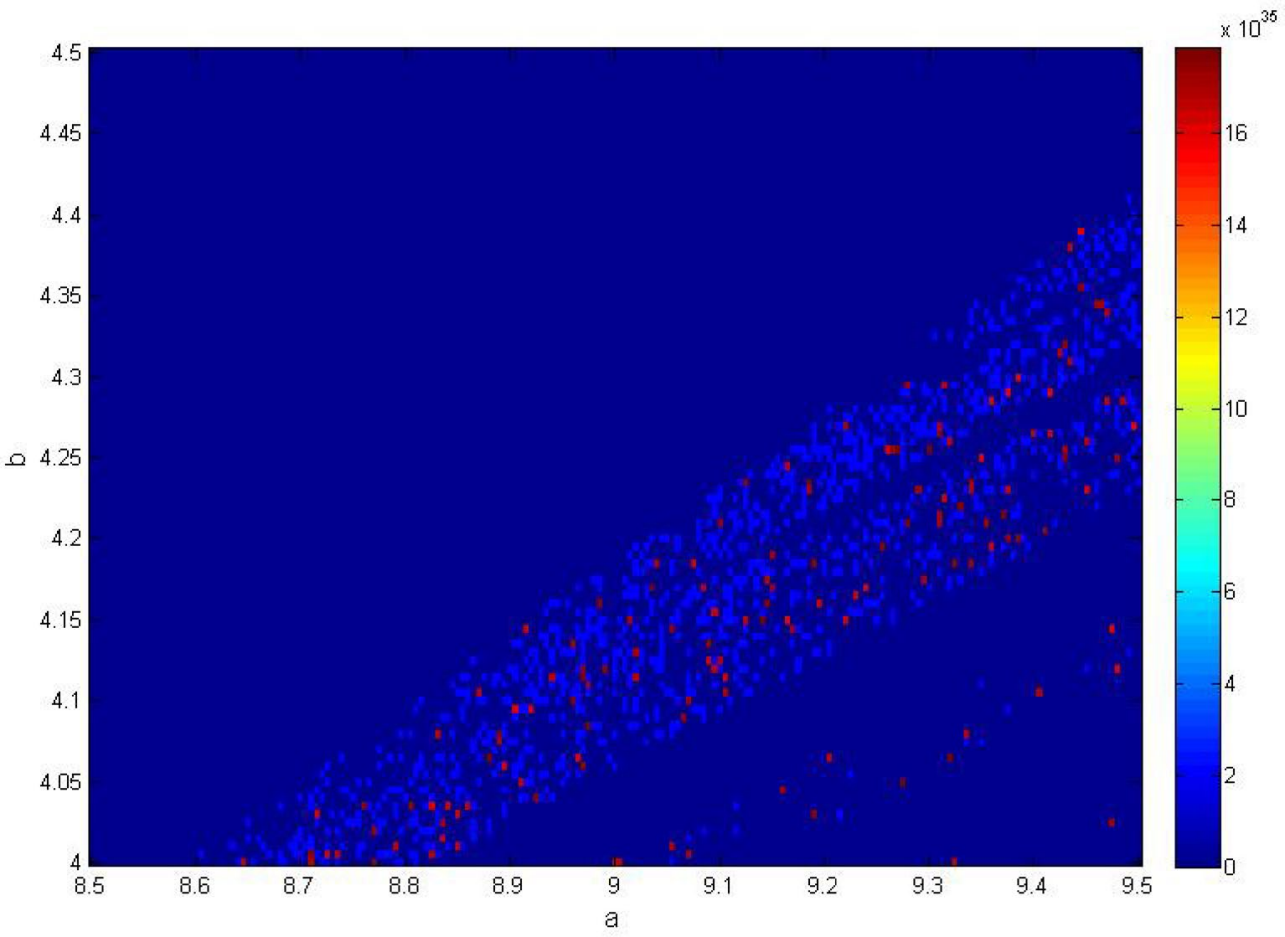
Two parameter Bifurcation diagram for the variation of parameters (**a**) and (**b**).

Figure 7 shows the two parameter bifurcation diagram for the variations of parameters *a* and *b*. The large dark-blue area of the diagram likely represents regions of stable or periodic behavior. This area represents the transition from stable/periodic behavior to chaotic dynamics as the parameters change. The complex, light blue region, suggests a wide range of dynamic behaviors, including chaos. The scattered nature of the colors in this area often reveals complex dynamics.

## Chaotic amplitude control

**Fig. 8:**
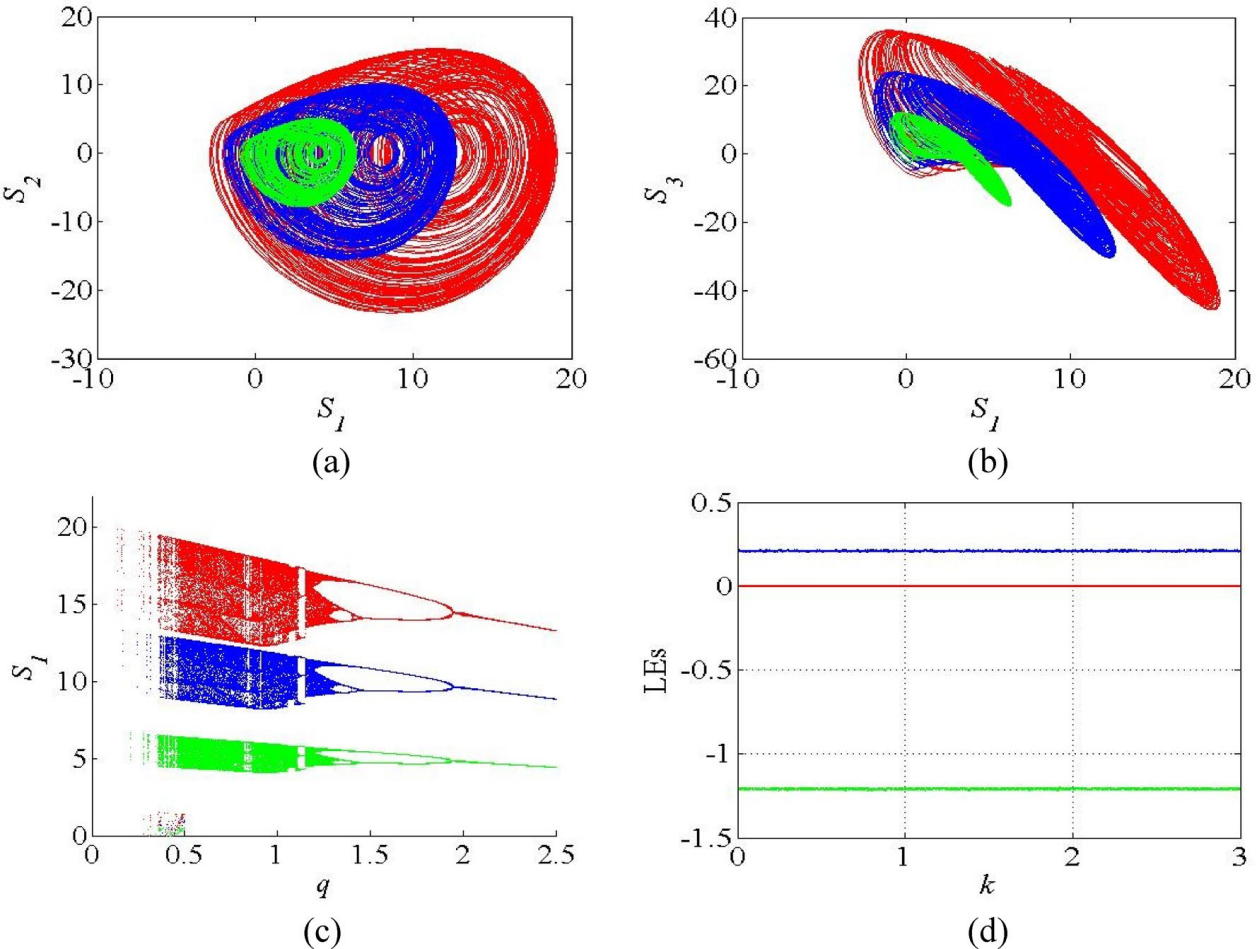
(**a**-**b**) Chaotic attractors of the CAC system (11); (**c**) Bifurcation diagrams with the scaling factor *k* = 1 (blue), *k* = 1.5 (red), *k* = 0.5 (green); (**d**) Constant LE spectrum against *k*.

Controlling the amplitude of chaotic signal is crucial for the various practical applications to maintain the reliable and safe operations^37^. In chaos based communication system, the amplitude control adjusts the amplitude level of chaotic signals to meet transmission requirements. In electronic based chaotic circuits, the uncontrolled signal fluctuations can exceed voltage or current limitation of electronic components, leading to permanent damage. The amplitude control ensures the safe operating ranges of electronic components for chaotic signals. In supply chain modelling, the amplitude control approach prevents the excessive fluctuations in inventory. The simple and efficient method to achieve the amplitude control in chaotic system is systematically scaling its state variables by the scaling factor. If amplitude of all the state variables is regulated, then the approach is known complete amplitude control. If we take ${S_1}=\frac{{{p_1}}}{k},{S_2}=\frac{{{p_2}}}{k},{S_3}=\frac{{{p_3}}}{k}$ in the original system (4), the modified system can be written as follows:

11 $$\begin{gathered} \dot{S}_{1} = S_{2} \hfill \\ \dot{S}_{2} = S_{3} \hfill \\ \dot{S}_{3} = aS_{1} - bS_{2} - S_{3} - S_{1}^{2} - c\sin (S_{1} ) - q|S_{1} | \hfill \\ \end{gathered}$$

where, *S*_1_, *S*_2_ and *S*_3_ are the state variables of the new modified system (11). Figure 8a-b shows the chaotic attractors of the modified system (11) with the scaling factor *k* = 1 (original), *k* = 1.5 (red), *k* = 0.5 (green).

It can be noticed from Fig. 8 that the proposed control approach amplifies the amplitude of the original system when *k* > 1 and reduces when *k* < 1, without disturbing its chaotic nature. In addition, we plotted the bifurcation diagrams as given in Fig. 8c, to verify the effectiveness of proposed control approach with the various values of scaling factor. It reveals that the scaling factor systematically oversees the bifurcation level without affecting its chaotic regions. Further, we plotted LE spectrum against the scaling factor as shown in Fig. 8d. Figure 8d reveals that the introduction of scaling factor does not affect the system’s sensitivity on initial conditions, evidenced by constant LE values thought the spectrum.

## Offset

Offset boosting control^38–40^ is used to shift the equilibrium points of the system’s attractor away from the origin without altering its complex dynamics. In cryptography and secure communication, the offset boosted attractors are used to represent different encoding scheme and multiple independent channels can be created, enhance the security. Offset boosting control allows moving from zero centered voltage, reducing DC interference. In the context of supply chain models, the offset boosting control the mean inventory level without suppressing natural fluctuations. In supply chains, shifts are routinely implemented through policy changes, and buffer inventory to absorb fluctuations and prevent disruptions while maintaining responsiveness. The key point is that offset boosting maintains the chaotic adaptability of the system while repositioning the operating point to a safer region of the state space, analogous to enhancing resilience without suppressing the system’s flexibility.

Offset boosting control is a simple but effective control method to shift the attractor of a chaotic system in phase space without modifying its chaotic dynamics. This control strategy shifts the mean value or base value of a particular chaotic signal. The offset boosting can be easily achieved by adding a constant term, known as the offset booster with any one or more states of the system. Importantly, the chaotic dynamics such as LE values and attractor geometry stay the same, only the position of the signal in phase space changes. In this work, first we implemented the offset boosting in *p*_2_ direction of the proposed system by adding booster parameter *b*_1_ with it. Equation (12) shows the modified system of (4) with the introduction of offset booster *b*1 in the *p*2 signal. Since the derivative of constant is zero, the booster parameter is added only in R.H.S of the system. The modified system (12) moves the position of attractor in *p*2 direction without disturbing its chaotic behavior. Analytical verification shows that the equilibrium points, Jacobin matrix and eigenvalues of the system (12) are identical to those of the original system (4). Therefore, the stability type of the system (12) is still same as the original system (4).

12 $$\begin{gathered} {{\dot {p}}_1}={p_2}+{b_1} \hfill \\ {{\dot {p}}_2}={p_3} \hfill \\ {{\dot {p}}_3}=a{p_1} - b({p_2}+{b_1}) - {p_3} - p_{1}^{2} - c\sin ({p_1}) - q|{p_1}| \hfill \\ \end{gathered}$$

**Fig. 9:**
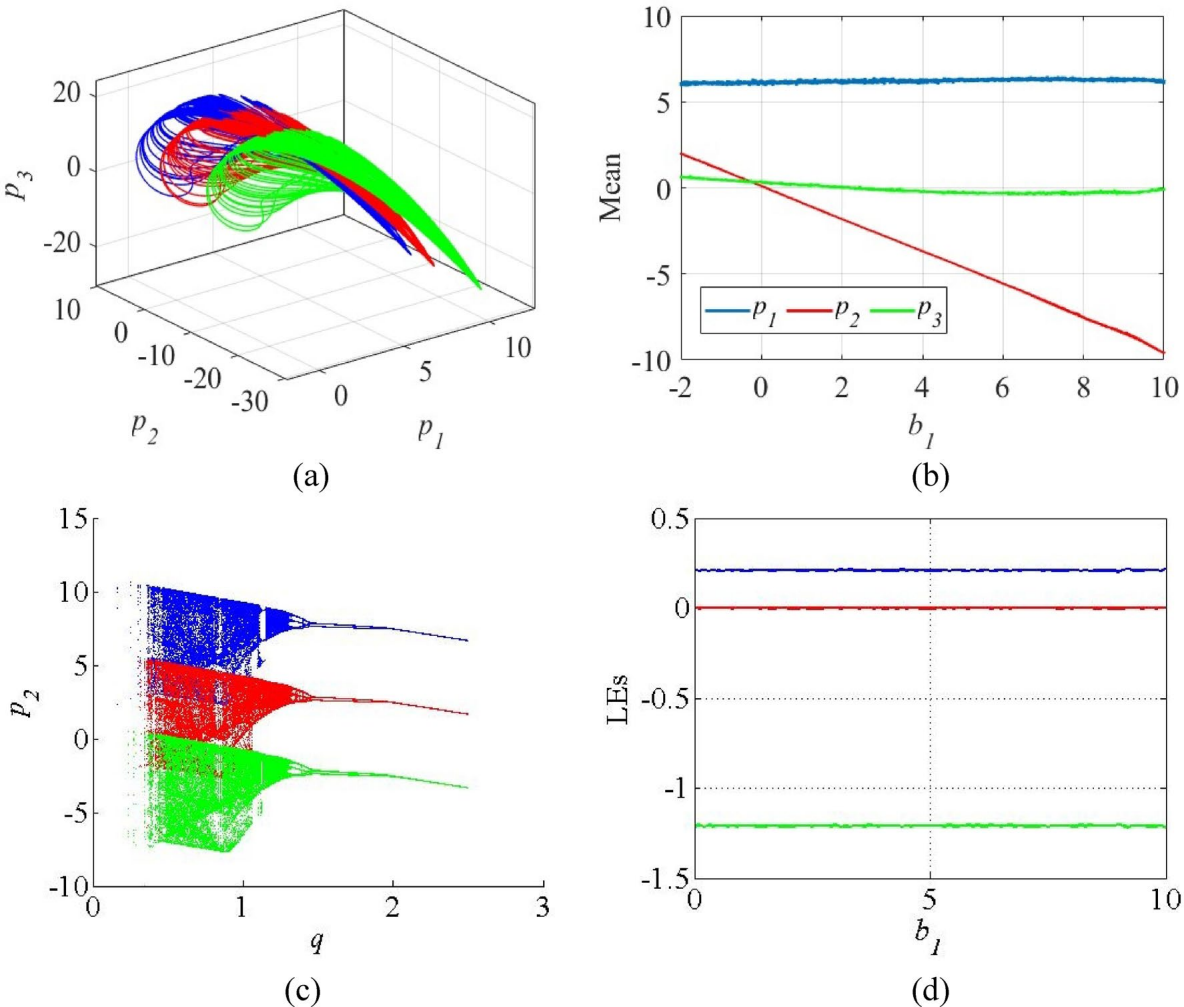
(**a**) Chaotic attractors of the system (12) with offset boosting along *p*_2_ direction; (**b**) Mean plots of the state variable against *b*_1_ (**c**) Bifurcation diagrams with the booster parameter *b*_1_ = 0 (blue), *b*_1_ = 5 (red), *b*_1_ = 10 (green); (**d**) Invariant LE spectrum against *b*_1_.

In Fig. 9, the effect of offset boosting control in the system (4) is illustrated through 3D phase space, mean plot, bifurcation diagram and LE plot. Figure 9a shows the chaotic attractor of the modified system (12) in 3D space when *b*_1_ = 0 corresponds to original system (shown in blue), *b*_1_ = 5 shown in red, *b*_1_ = 10 shown in green. The positive values of offset booster cause the chaotic signal *p*_2_ to shift in negative directions. Figure 9b shows the relationship between of mean values of the chaotic signal and the booster parameter *b*_1_. The plot shows that only the mean value of *p*_2_ changes linearly with the offset booster *b*_1_, while the mean values of other state variables remain constant. The bifurcation diagrams of the system (12) for the parameter *q* against the state variable *p*_2_ are given in Fig. 9c in which the booster parameter shifts the bifurcation without disturbing its chaotic regions. Further, the LE spectrum of the system (12) against the parameter *b*_1_ is given in Fig. 9d which indicate that the parameter *b*_1_ shifts the chaotic signal without modifying the LE values of the original system (4).

Next, we implemented the offset boosting in *p*_3_ direction of the proposed system by adding booster parameter *b*_2_ with in it. Equation (13) shows the modified system of (4) with the introduction of offset booster *b*_2_ in the *p*_3_ signal. The modified system (13) moves the position of attractor in *p*_3_ direction without disturbing its chaotic behavior and stability type.

13 $$\begin{gathered} {{\dot {p}}_1}={p_2} \hfill \\ {{\dot {p}}_2}={p_3}+{b_2} \hfill \\ {{\dot {p}}_3}=a{p_1} - b{p_2} - ({p_3}+{b_2}) - p_{1}^{2} - c\sin ({p_1}) - q|{p_1}| \hfill \\ \end{gathered}$$

**Fig. 10:**
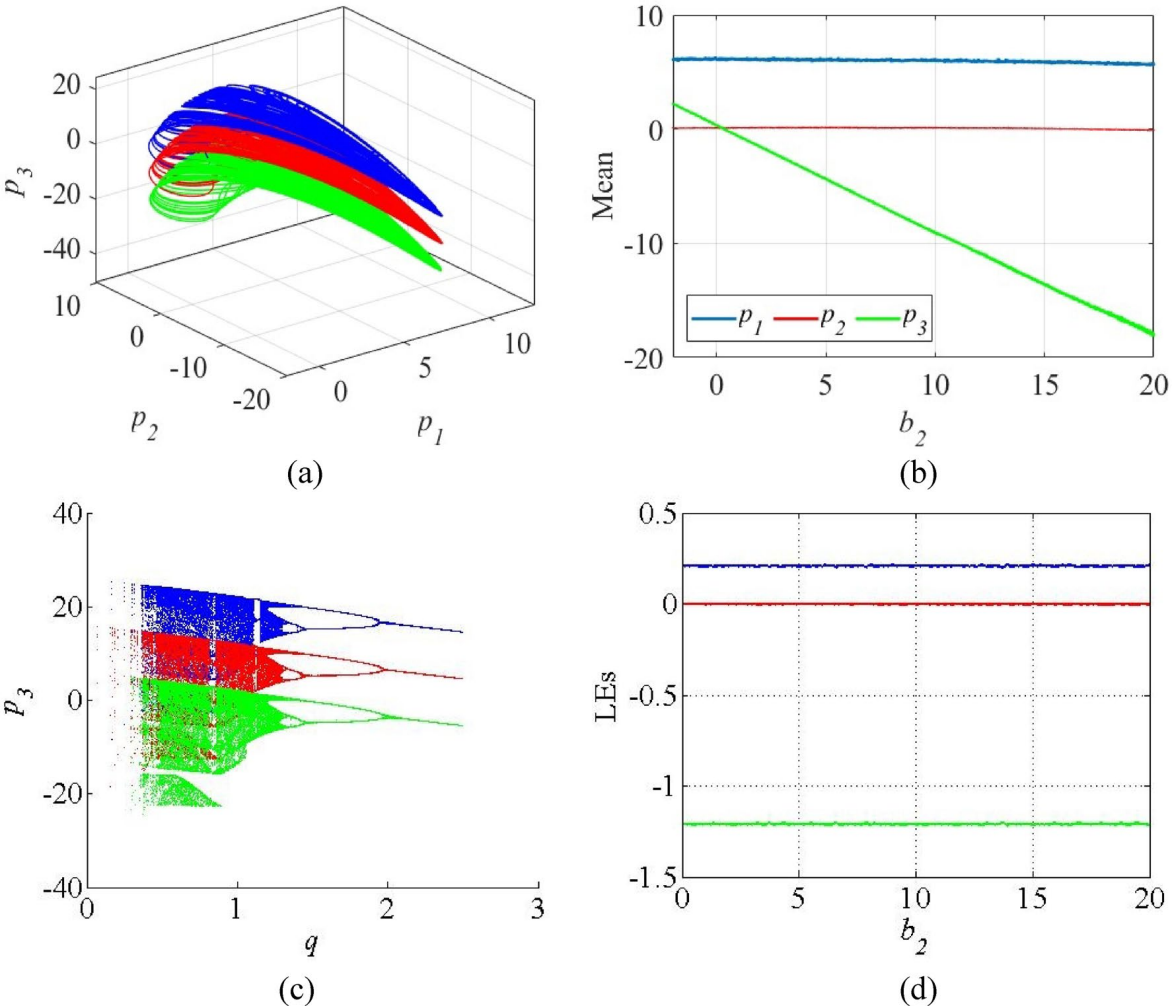
(**a**) Chaotic attractors of the system (13) with offset boosting along *p*_3_ direction; (**b**) Mean plots of the state variable against *b*_2_ (**c**) Bifurcation diagrams with the booster parameter *b*_2_ = 0 (blue), *b*_2_ = 10 (red), *b*_2_ = 20 (green); (**d**) Invariant LE spectrum of *b*_2_.

Figure 10 shows the results of the proposed offset boosting methodology in *p*_3_ direction. The chaotic attractors of the system (13) with *b*_2_ = 0 (blue), *b*_2_ = 10 (red), *b*_2_ = 20 (green) are shown in Fig. 10a. Figure 10b shows the variation of mean values of state variables against the control parameter *b*_2_. As the control parameter increases, the mean value of *p*_3_ reduces with *b*_2_ in linear manner, while keeping the mean values of other state variables constant. The bifurcation diagrams of the system (13) for the parameter *q* against the state variable *p*_3_ are given in Fig. 10c in which the booster parameter shifts the bifurcation without disturbing its chaotic regions. Figure 10d, the invariant LE spectrum against the parameter *b*_2_ indicates that the proposed control methodology does not affect the system’s sensitivity on initial conditions behavior.

## Results and discussion

- The proposed chaotic three-tier supply chain model incorporates absolute and sinusoidal nonlinearities into the Hamidzadeh framework, providing a realistic representation of structured, periodic uncertainties such as seasonal demand fluctuations and production cycles. The complexity of the proposed system is notably improved in terms of Lyapunov exponent values *l*_1_ = 0.2121, *l*_2_ = 0, *l*_3_ = -1.2121 and Lyapunov dimension *D*_*L*_ = 2.1750, compared with the existing systems in literature.
- The dynamical analysis using one parameter and two parameter bifurcation diagrams, Lyapunov exponent spectrum and phase projections demonstrates increased sensitivity to initial conditions, and broader chaotic parameter ranges.
- The rescaling based amplitude control method effectively regulates the amplitude of all the state variables in the proposed system. The offset boosting using a bias term effectively relocate the attractors while preserving the chaotic dynamics. These strategies have practical analogs in supply chain operations, such as adjusting inventory or production baselines, allowing networks to maintain resilience and adaptability under fluctuating conditions.

## Conclusion

In this study, a novel chaotic three-tier supply chain system was developed by integrating absolute function and sinusoidal nonlinearities into the established Hamidzadeh framework. The proposed model exhibited richer dynamical characteristics, as confirmed through Lyapunov exponent analysis, Lyapunov dimension evaluation, and bifurcation studies. The computed Lyapunov exponents, *l*_1_ = 0.2121, *l*_2_ = 0, *l*_3_ = -1.2121, the maximum Lyapunov exponent, *L*_max_ = 0.212 and Lyapunov dimension *D*_*L*_ = 2.1750 are significantly higher than the similar existing models, indicating the higher sensitivity on initial conditions and a greater degree of chaos. The incorporation of amplitude control and offset boosting provided effective means to regulate the system’s chaotic responses without compromising their intrinsic complexity, offering flexibility for practical applications where both adaptability and stability are required. Comparative analysis with existing models demonstrated that the proposed system achieves higher chaotic intensity and greater controllability, making it a valuable contribution to both theoretical research and applied supply chain management. Beyond advancing the modeling of supply chain dynamics, the results underscore the potential of combining nonlinear system theory with targeted control strategies to design more resilient, adaptive, and efficient supply chain networks in the face of uncertainty. Future research could extend this framework to fractional-order dynamics, hybrid control schemes, or integration with real-world supply chain data for validation and implementation.

## Data Availability

The data that support the findings of this study are available from the corresponding author on reasonable request.
